# Synergistic effects of combined hypnotic drugs on sleep in mice

**DOI:** 10.3389/fphar.2025.1596813

**Published:** 2025-07-23

**Authors:** Rui Bai, Chu Wang, Fanghui Wu, Yifeng Yin, Yanli Liu, Yuan Gao, Yunyang Song

**Affiliations:** ^1^College of Life Science and Technology, Gansu Agricultural University, Lanzhou, Gansu, China; ^2^ State Key Laboratory of NBC Protection for Civilian, Beijing, China; ^3^Department of Human Anatomy, Baotou Medical College, Inner Mongolia University of Science and Technology, Baotou, Inner Mongolia, China; ^4^ Gansu Key Laboratory of Animal Generational Physiology and Reproductive Regulation, Lanzhou, Gansu, China

**Keywords:** sleep, hypnotics, dexmedetomidine, eszopiclone, combination, synergistic effects

## Abstract

**Study objectives:**

To investigate the interaction and potential mechanisms of the combined administration of dexmedetomidine (DMED) and eszopiclone (ESZ) on sleep in mice.

**Methods:**

The effects of DMED, ESZ and the combination of the two drugs on the sleep status of mice were investigated by using the loss of righting reflex (LORR) as an indicator, the sleep-related parameters were recorded, and the interactions of the combination of DMED and ESZ on sleep were determined by isobolographic analysis. The effects of DMED and ESZ on sleep structure, the regulation of c-Fos protein expression in the ventrolateral preoptic area (VLPO) and the tuberomammillary nucleus (TMN) and the regulation of neurotransmitter levels were further investigated by combining electroencephalogram/electromyogram (EEG/EMG) sleep phase analysis, c-Fos immunofluorescence, and neurotransmitter content measurements in the brain.

**Results:**

Isobolographic analysis demonstrated that the combination of DMED and ESZ had a synergistic effect on sleep in mice. The duration of non-rapid eye movement (NREM) sleep was significantly prolonged; the number of c-Fos positive neurons increased in the VLPO and decreased in the TMN; the levels of excitatory neurotransmitters were reduced, while the levels of inhibitory neurotransmitters were elevated.

**Conclusion:**

This study reveals that the combination of DMED and ESZ exerts a synergistic effect on sleep in mice. The underlying mechanism may involve the prolongation of NREM sleep, modulation of neuronal activity in the brain regions of VLPO and TMN, as well as alteration in the levels of brain neurotransmitters.

## Graphical Abstract

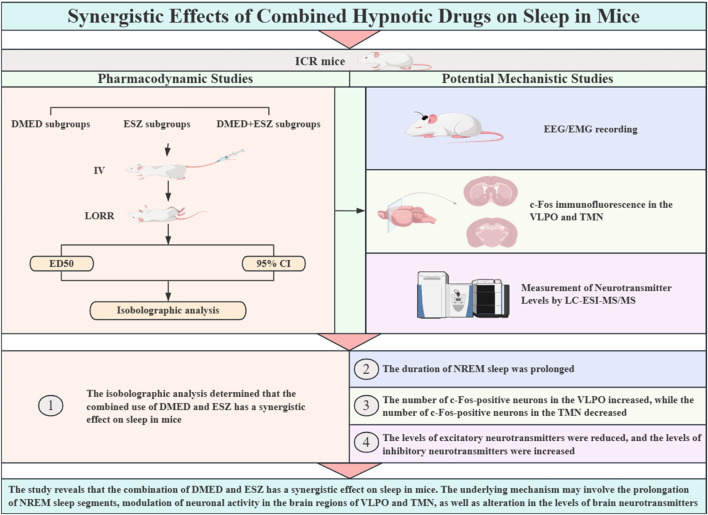

## 1 Introduction

Sleep is a systemic physiological phenomenon in the biological world (Tononi et al., 2024), which can reduce the decomposition of body energy, promote the removal of metabolic wastes from the brain, and play a crucial role in maintaining wakefulness, cognition, learning, and memory. It also has an important impact on regulating the secretion of physiological hormones and the immune function of the body (Baranwal et al., 2023). Normal sleep phases can be divided into non-rapid eye movement (NREM) sleep and rapid eye movement (REM) sleep, with distinct electroencephalogram (EEG) and behavioral characteristics in different states. NREM sleep plays a key role in physical recovery, energy reserve, and memory consolidation, while REM sleep is associated with dreaming and cognitive processing (Sun et al., 2020). The sleep-wake state is regulated by the brain’s neurotransmitter systems. The wake-promoting pathways involve monoaminergic neurons in the anterior brainstem and caudal hypothalamus that directly project to the cerebral cortex, hypothalamus, and thalamus to regulate wakefulness. These monoaminergic neurons include norepinephrineergic neurons in the locus coeruleus (LC) region (Bajwa and Kulshrestha, 2013), serotonergic neurons in the dorsal raphe nucleus (DRN) (Oikonomou et al., 2019), dopaminergic neurons in the ventral tegmental area (VTA) (Montero et al., 2021), and histaminergic neurons in the tuberomammillary nucleus (TMN) (Scammell et al., 2019). These central neurons generate wakefulness through simultaneous stimulation of cortical activation and an increase in muscle tone, with their firing frequency highest during the awake state and decreasing during NREM sleep. Additionally, cholinergic neurons in the laterodorsal tegmental nucleus (LDT) and pedunculopontine nucleus (PPT) are the most critical components of the ascending arousal system (Jones, 2020), while glutamatergic neurons mediating amino acid neurotransmission drive wakefulness in the thalamocortical circuit. During NREM sleep, gamma-aminobutyric acidergic (GABAergic) neurons in the ventrolateral preoptic nucleus (VLPO) of the anterior hypothalamus inhibit wakefulness and arousal via direct projections to the histaminergic TMN. However, with the continuous development of society, factors such as the accelerated pace of life and heightened stress levels have contributed to the widespread occurrence of sleep disorders. These disorders are typically manifested as difficulty falling asleep, maintaining sleep, frequent awakenings, or early morning awakenings, which result in decreased daytime functioning and significantly disrupt individuals’ daily lives and occupational performance (Hughes et al., 2021). Currently, cognitive behavioral therapy (CBT) is the first-line treatment for insomnia disorders, but due to limitations including protracted treatment duration and delayed therapeutic onset, sedative-hypnotic drugs remain the preferred intervention for most insomnia patients. Nevertheless, the medication patterns for sleep disorders are relatively homogeneous, characterized by low efficacy and high side effects. Long-term application can disrupt the brain’s reward system, leading to addictive behaviors and subsequently causing cognitive dysfunction (Crowe and Stranks, 2018; He et al., 2019). It can also interfere with the normal function of neurotransmitters, thereby affecting mood regulation (Ciucă Anghel et al., 2023). Additionally, it significantly increases the risk of developing neurological diseases (He et al., 2019; Zheng et al., 2023). Therefore, it is essential to conduct studies on drug combinations for sleep regulation to minimize the potential adverse effects associated with the use of single drugs at high doses.

Dexmedetomidine (DMED), a highly selective α_2_-adrenergic receptor (α_2_-AR) agonist, exerts pharmacological effects by acting on α_2_-AR in the central nervous system and the periphery (Chima et al., 2022), Its effects include sedation, analgesia, hypnosis, the antisympathetic effects, anxiolysis, delirium reduction, anti-inflammatory properties, and protection of vital organs. In recent years, DMED has been increasingly used in the treatment of chronic insomnia (Ramaswamy et al., 2021). It is generally believed that DMED induces sleep by activating presynaptic α_2_ receptors on norepinephrine (NE) neurons in the LC, thereby reducing NE release through a G_i_-coupled mechanism (Fan et al., 2023). Studies have also demonstrated that DMED exerts sleep effects by activating GABAergic neurons in the lateral preoptic area (LPO) (Zhang et al., 2015). Compared with other sedative drugs, the sleep induced by DMED is similar to physiologic sleep, with no respiratory depression and increased sleep depth (Wu et al., 2016; Ren et al., 2018; Bao et al., 2021; Dong et al., 2022). At the same time, patients can be easily awakened when DMED exerts its sedative effect. Although the neural mechanism underlying this rapid arousability during DMED sedation remains unclear and may involve VTA dopaminergic neurons (Qiu et al., 2020), DMED is widely used in clinical anesthesia and intensive care unit (ICU) sedation due to this unique property (Wang et al., 2023; Duan et al., 2024). In addition, several studies have shown that DMED can prolong the total duration of NREM, increase the depth of sleep, and improve the quality of sleep (Feng et al., 2018; Fan et al., 2023). However, DMED has some limitations. It may trigger cardiovascular side effects such as hypertension, bradycardia, and hypotension caused by postsynaptic α_2_-AR activation (Weerink et al., 2017), as well as dose-dependence risk, possible tolerance and withdrawal reactions with long-term use, and higher economic costs, all of which limit its use in clinical practice to some extent.

Eszopiclone (ESZ), as a third-generation novel non-benzodiazepine drug (NBZD), belongs to the pyrrolidine derivatives of the cyclopyrrolone family (Pinto et al., 2016), and is a GABA receptor agonist, capable of selective binding to GABA-A receptors coupled to benzodiazepine receptors, enhancing the inhibitory effect of GABA on neurons and exerting anxiolytic, sedative, and hypnotic effects (Hanson et al., 2008). Additionally, studies have demonstrated that inhibition of hypocretin (HCRT) neurons in the lateral hypothalamus (LH), alongside suppression of basal forebrain and DRN, represents a potential mechanism underlying the sleep-promoting effects of ESZ. ESZ typically induces sleep, which is characterized by a shorter NREM latency, prolonged NREM sleep duration, and unchanged REM sleep duration (Gerashchenko et al., 2017). It facilitates NREM sleep by enhancing GABAergic transmission in the thalamic reticular nucleus, thereby inducing sleep spindle generation (Wamsley et al., 2013; Dimitrios et al., 2020). ESZ has the advantages of rapid onset of action, short half-life, and few side effects, especially in improving sleep maintenance disorders. Therefore, ESZ is commonly used in the treatment of insomnia and can effectively improve sleep onset and maintenance in patients. Despite the significant advantages of ESZ in the treatment of insomnia, there are some limitations and application drawbacks. It may cause common adverse effects, such as dry mouth, dizziness, fatigue, abnormal taste, and daytime sleepiness (Liang et al., 2019), and long-term use may produce drug dependence and withdrawal reactions. In addition, the tolerability and higher economic cost issues of ESZ have limited its use in clinical settings.

Although DMED and ESZ each have well-defined roles and advantages in regulating sleep, studies of their combined use have not been reported. Since DMED mainly acts in regions such as the brainstem and hypothalamus, it regulates the noradrenergic system by agonizing α_2_-AR, which in turn inhibits sympathetic activity to induce sleep (Nelson et al., 2003), whereas ESZ mainly acts in regions such as the cerebral cortex, the limbic system, and the thalamus, and promotes sleep by augmenting GABAergic inhibitory neurotransmission, and decreasing the excitability of the cortex and the limbic system (Hanson et al., 2008). The two are complementary in their sites of action and neurotransmitter regulatory pathways, and their combined application may further enhance sleep depth and improve sleep quality. Therefore, the combination is expected to enhance the therapeutic effect while reducing the dose of a single drug, decrease the occurrence of side effects, and optimize the economic cost of treatment.

For this reason, the present study focused on the combination of DMED and ESZ as an entry point, integrating isobolographic analysis, electroencephalogram/electromyogram (EEG/EMG) sleep monitoring, c-Fos immunofluorescence staining, and liquid chromatography-electrospray ionization-tandem mass spectrometry (LC-ESI-MS/MS) to investigate the interaction between these two drugs on sleep in mice and their potential mechanisms of action. Our results demonstrate that the combination of DMED and ESZ exhibits a synergistic effect on sleep in mice. The underlying mechanism may involve prolonging NREM sleep, modulating neuronal activity in the VLPO and TMN brain regions, and altering brain neurotransmitter levels. These findings provide a robust theoretical basis for clinical combination therapy to improve sleep quality.

## 2 Materials and methods

### 2.1 Experimental animals

Healthy male ICR mice, SPF grade, 6–8 weeks old, weighing 18–20 g, were purchased from Beijing HFK Bioscience Co., Ltd. (Beijing, China). The animals were housed in groups of 4 per cage under controlled conditions: temperature 22°C ± 2°C, humidity 50% ± 10%, and a 12 h/12 h light-dark cycle (light on/off time: 08:00/20:00). Standard feed and water were provided *ad libitum*. The animal welfare and experimental project were approved by the Animal Ethics Committee of State Key Laboratory of NBC Protection for Civilian, and every effort was made in the experiment to reduce the number of animals used and any pain or discomfort they might experience.

### 2.2 Drugs, reagents, and instruments

Dexmedetomidine (315988, Hebei Pinkeyan Biotechnology Co., Ltd., China); Eszopiclone (2410892Z-YT-01, Standard Drug Group Co., Ltd., China); Pentobarbital sodium (63-06-01, Shanghai Chemical Reagent Procurement and Supply Station, China); Triton X-100 (30188928, Sinopharm Group, China); OCT embedding agent (Sakura Finetek, United States); Rabbit anti-c-Fos antibody (ab222699, Abcam, United States); Goat anti-rabbit secondary antibody (A32740, Thermo Fisher Scientific, United States). Dopamine (Z21J10R91113, Shanghai Yuanye Bio-Technology Co., Ltd., China); Norepinephrine (H01M10C81693, Shanghai Yuanye Bio-Technology Co., Ltd., China); 5-Hydroxytryptophan (111656–200401, National Institutes for Food and Drug Control, China); 5-Hydroxyindole-3-Acetic Acid (21A020-I9, SHANGHAI ZZBIO CO., LTD., China); γ-Aminobutyric acid (Z07J10H79273, Shanghai Yuanye Bio-Technology Co., Ltd., China); Acetylcholine (Z08N8H47718, Shanghai Yuanye Bio-Technology Co., Ltd., China); Histamine(X17S9B70455, Shanghai Yuanye Bio-Technology Co., Ltd., China); Glutamic acid (S12A10I85582, Shanghai Yuanye Bio-Technology Co., Ltd., China); HPLC grade-acetonitrile (A998-4, Thermo Fisher Scientific, United States); HPLC grade-methanol (A452-4, Thermo Fisher Scientific, United States); formic acid (UN 1779, CNW Technologies GmbH, Germany); LCMS grade-purified water (W6-4, Thermo Fisher Scientific, United States).

Leica cryostat (CM 1950, Leica Biosystems, Germany); Confocal microscope (Stellaris5, Leica Biosystems, Germany); Circuit board soldering iron (SBK936B, BAKON Electronic Technology Co., Ltd., China); Miniature cranial drill (RWD Life science Co., Ltd., China); Stereotaxic apparatus (71000-M, RWD Life science Co., Ltd., China); Small animal sleep analysis and detection system (8200-K1, Pinnacle Technologies Co., Ltd., United States); Liquid chromatography-mass spectrometer (QTRAP 6500+, AB SCIEX); Cryo-mill (Wonbio-96E, Shanghai Wanbai Biotechnology Co., Ltd., China); Ultrasonic Cleaner (SBL-10DT, NingBo Scientz Biotechnology Co., Ltd., China); Freezing Centrifuge Concentration Dryer (LNG-T98, Taicang Huamei Biochemical Instrument Factory, China).

### 2.3 Pharmacodynamic study of DMED and ESZ alone

Grouping and administration: ICR mice aged 6–8 weeks were selected for the study and divided into a control group (Control group) and experimental groups (DMED group, ESZ group, and DMED+ESZ group) using the random number table method. Each experimental group was further divided into 5 subgroups according to the different drug doses, and different doses of drugs, respectively, were given by intravenous (IV) injection via the caudal vein with a volume of 0.1 mL/10 g.

Preparation of drugs for IV injection: DMED was dissolved in 100% dimethyl sulfoxide (DMSO) to prepare a 2 mg/mL stock solution, which was aliquoted and stored at −20°C to preserve stability. Before intravenous injection, the stock solution was further diluted with 0.9% saline to achieve the desired dose. The final DMSO concentration in the injected solution was ≤1% (v/v), ensuring biocompatibility and minimal solvent-related toxicity. ESZ was dissolved in 50 mM sodium acetate buffer (pH 4.5 ± 0.1) to prepare a 20 mg/mL stock solution, aliquoted, and stored at −20°C to maintain stability. The stock solution was diluted with 0.9% sterile saline immediately prior to intravenous administration to achieve desired concentrations.

Determination of administration dose: Mice in each experimental group were subjected to the loss of righting reflex (LORR) experiment. After the administration of the drug, the mice were gently placed in the cage in the supine position, and the mice were turned over once every 1 min; if they remained in the supine position for ≥1 min, it was determined as LORR; if the mice were restored to the prone position for ≥2 times in 1 min, it was judged as recovery of the turning-right reflex (Revel et al., 2009). The LORR was used as the criterion to obtain the minimum dose (ED_100_) with a 100% response rate to DMED and ESZ hypnosis and the maximum dose (ED_0_) with a 0% response rate in mice, respectively. Within this dose range, it was divided into 5 dose groups in an equiproportional series, and the common ratio (r) of the doses of each group was r = $\sqrt[{5-1}]{\text{ED}100/\text{ED}0}$ , and after obtaining r, the dose of the next adjacent group was obtained by multiplying r from the first dose group (ED_0_), that is to say, the doses of each group were ED_0_, r·ED_0_, r^2^·ED_0_, r^3^·ED_0_, r^4^·ED_0_, r^5^·ED_0_.

The sleeping rate, sleep latency, and total sleep time were recorded for each group of mice following drug administration. The ED_50_ and its 95% confidence interval (CI) of DMED and ESZ alone were calculated using the Bliss method.

### 2.4 Pharmacodynamic study of DMED and ESZ in combination

After obtaining the respective median effective doses (ED_50_) of DMED and ESZ, fixed-dose combinations were administered based on the ratio of DMED’s ED_50_ to ESZ’s ED_50_ (DMED’s ED_50_:ESZ’s ED_50_). The experimental methodology followed the same procedures as described for individual drug administration. Isobolographic analysis was employed to assess the interaction between the two drugs. The ED_50_ of DMED in the combination and its 95% CI were plotted on the x-axis, while the ED_50_ of ESZ and its 95% CI were plotted on the y-axis. A line connecting the two ED_50_ points was drawn to represent the additive effect line, and the 95% CI lines were connected to establish the 95% CI of the additive line. The ED_50_ of the combined treatment was then plotted on the same coordinate axis. If the ED_50_ of the combined treatment fell on the additive line or within its 95% CI, the interaction was considered additive. If it fell to the left of the additive line and its 95% CI, the interaction was synergistic. Conversely, if it fell to the right of the additive line and its 95% CI, the interaction was antagonistic.

### 2.5 Burial of EEG/EMG

Mice were anesthetized via intraperitoneal injection of 1% sodium pentobarbital (70 mg/kg body weight). The heads and necks of the mice were shaved and securely positioned in a brain stereotaxic apparatus. EEG and EMG electrodes were surgically implanted into the skull following a previously established protocol (Sharma et al., 2018), small holes were drilled into the skull, and stainless steel screws were inserted as EEG electrodes. The entire device was then anchored to the skull using dental cement. After the cement had fully cured, two insulated silver electrode wires for EMG recording were inserted bilaterally into the trapezius muscles of the neck. Residual cement around the surgical site was carefully removed, and the incision was closed with sutures. The mice were placed in a lateral recumbent position on a 37°C temperature-controlled heating pad to recover until fully conscious. Experimental recordings were conducted 1-week post-surgery to allow for postoperative recovery.

### 2.6 EEG/EMG data recording and processing

Twenty-four mice exhibiting normal EEG and EMG signal patterns were selected and randomly assigned to four groups (*n* = 6 per group): Control group, DMED group, ESZ group, and DMED+ESZ group. Each mouse was placed in a sleep-monitoring chamber for continuous sleep-stage data acquisition. EEG and EMG recordings commenced at 08:00 and lasted for 12 h. The 12-h EEG and EMG voltage traces were segmented into 4,320 epochs, with each epoch lasting 10 s. Power thresholds for EEG and EMG were defined for each epoch based on energy values (criteria detailed in Tables 1, 2). The recorded data were exported and processed using Sirenia Acquisition 2.1.5 software to generate voltage-time plots for individual mice. These plots were subsequently analyzed in Sirenia Sleep 2.1.5 software to quantify the duration of distinct sleep-wake states, including wakefulness (Wake), non-rapid eye movement (NREM) sleep, and rapid eye movement (REM) sleep. Additionally, graphical representations of state transitions were generated to visualize temporal changes between these states.

**TABLE 1:** EEG power analysis definition rules.

Name	Low frequency	High frequency
Full	0	1,000
Alpha	8	13
Beta	13	30
Gamma	35	44
Delta	0.5	4
Theta	5.5	8.5

**TABLE 2:** EMG power analysis definition rules.

Name	Low frequency	High frequency
Full	0	1,000
10 ~ 50	10	50
50 ~ 100	50	100
100 ~ 150	100	150
150 ~ 200	150	200
50 ~ 150	50	150

### 2.7 Preparation of brain tissue and frozen section

All experimental mice were deeply anesthetized via intraperitoneal injection of 1% sodium pentobarbital (70 mg/kg body weight) 1.5 h after drug administration. Following anesthesia, a thoracotomy was performed to expose the heart for transcardial perfusion fixation. Perfusion was conducted sequentially: first with 30 mL of phosphate buffered saline (PBS, 0.01 M, pH 7.4) to clear intravascular blood, followed by 30 mL of 4% paraformaldehyde (PFA) solution for fixation. The fresh brain tissues were fixed in 4% PFA solution for 24 h at 4°C and then dehydrated sequentially through a gradient of 10%, 20%, and 30% sucrose solution, with replacement to a higher concentration signified by the tissues sinking to the bottom. Once they had sunk to the bottom in 30% sucrose solution, they were ready for frozen sectioning. For this experiment, OCT (Optimal cutting temperature compound) embedding was used, and continuous coronal sectioning was carried out using a Leica cryostat with a section thickness of 30 µm.

### 2.8 c-Fos immunofluorescence

Sections were treated with 3.0% hydrogen peroxide for 25 min and rinsed with phosphate-buffered saline containing 0.1% Tween-20 (PBST). Sections were then blocked using bovine serum albumin (BSA) containing 0.3% Triton x-100 for 2 h at 4°C. After rinsing, sections were incubated with rabbit-derived primary anti-c-Fos antibody (1:1000 in PBS) overnight at 4°C. After rinsing, sections were placed with goat anti-rabbit secondary antibody (1:500 in PBS) for 1 h at 37°C, were dropwise added with anti-fade mounting medium (with DAPI), and then sealed with a cap. The stained sections were observed under a confocal microscope at ×20 and ×40 magnification. Cells with immunofluorescence intensity exceeding twice the background level were classified as c-Fos positive. Three brain sections containing the target nuclei were counted, and the number of c-Fos positive cells in the VLPO and TMN was quantified using ImageJ software with the Cell Counter plugin.

### 2.9 Neurotransmitter standard solution preparation and sample processing

Accurate amounts of standard compounds, including norepinephrine (NE), dopamine (DA), 5-hydroxytryptamine (5-HT), 5-hydroxyindoleacetic acid (5-HIAA), γ-aminobutyric acid (GABA), histamine (His), acetylcholine (Ach), and L-glutamic acid (Glu), were weighed and dissolved in methanol or water to prepare individual stock solutions. Appropriate volumes of each stock solution were mixed to create a combined standard solution, which was then diluted with water to the desired concentration to prepare the working standard solution. Isotope-labeled standards (Trp-D5 and Glu-13C5) were also accurately weighed and dissolved in methanol to prepare individual stock solutions. These isotope-labeled stock solutions were then mixed and diluted with water to prepare an isotopic internal standard mixture at concentrations of 5,000 ng/mL and 1,000 ng/mL.

Following transcardial perfusion, the whole brain was rapidly dissected for further experiments. The dissected brain tissues were placed into 2 mL EP tubes. Steel beads, 20 μL of isotope internal standard (5,000 ng/mL), and 480 μL of 80% methanol aqueous solution were added to the tubes. The samples were homogenized using a tissue lyser, incubated at −20°C for 30 min, and then centrifuged at 14,000 *g* for 15 min at 4°C. A total of 250 μL of the supernatant was transferred to a new tube and dried by centrifugal evaporation. The dried residue was resuspended in ultrapure water via vortexing, followed by centrifugation at 14,000 × g for 5 min. The resulting supernatant was then transferred to a vial for HPLC-MS/MS analysis to obtain the low-concentration test sample. For the high-concentration sample, 5 μL of the original supernatant was mixed with 245 μL of ultrapure water by vortexing. Subsequently, 50 μL of this diluted solution was combined with 50 μL of isotope internal standard (1,000 ng/mL). After thorough vortexing and centrifugation at 14,000 × g for 5 min, the supernatant was transferred to a separate vial, yielding the high-concentration test sample.

### 2.10 Determination of neurotransmitter levels

LC-ESI-MS/MS was used to quantify the target compounds in the samples. Chromatographic conditions: ExionLC AD system, Waters HSS T3 (2.1 × 100 mm, 1.8 μm) liquid chromatography column, column temperature 35°C, injection volume 1 μL. Mobile phase A was 0.1% formic acid in water, and mobile phase B was 0.1% formic acid in acetonitrile The chromatographic gradients are shown in Table 3. Mass spectrometry conditions: AB SCIEX QTRAP 6500+ with positive/negative mode detection, Curtain Gas (CUR) of 35 psi, Collision Gas (CAD) of Medium, IonSpray Voltage (IS) of +5,500/−4,500 V, Temperature (TEM) of 550°C, Ion Source Gas1 (GS1) is 55 psi, and Ion Source Gas2 (GS2) is 55 psi. Default parameters were used in the AB Sciex quantitative software OS for the automatic identification and integration of each ionic fragment with the aid of manual checking. A linear regression standard curve was plotted using the ratio of the mass spectral peak area of the analyte to the peak area of the internal standard as the vertical coordinate and the concentration as the horizontal coordinate. To calculate the sample concentration: The ratio of the mass spectral peak area of the sample analyte to the peak area of the internal standard is substituted into the linear equation to calculate the concentration result.

**TABLE 3:** Chromatographic gradients.

Time (min)	Flow rate (mL/min)	A%	B%
0.00	0.30	100.0	0.0
1.00	0.30	100.0	0.0
3.00	0.30	95.0	5.0
5.00	0.30	90.0	10.0
6.00	0.30	85.0	15.0
7.00	0.30	85.0	15.0
10.00	0.30	40.0	60.0
11.00	0.30	0.0	100.0
12.00	0.30	0.0	100.0
12.01	0.30	100.0	0.0
13.00	0.30	100.0	0.0
15.00	0.30	100.0	0.0

### 2.11 Statistics and analysis of data

The experimental data were presented as Mean ± SEM. Statistical analyses were performed using GraphPad Prism version 9.1.0 for Windows (GraphPad Software, La Jolla, CA, United States). One-way analysis of variance (one-way ANOVA) followed by Bonferroni’s *post hoc* test for multiple comparisons was used to analyze the data. The significance threshold was set at p < 0.05.

## 3 Results

### 3.1 Dose-effect relationship between DMED and ESZ alone on sleep in mice

**FIGURE 1:**
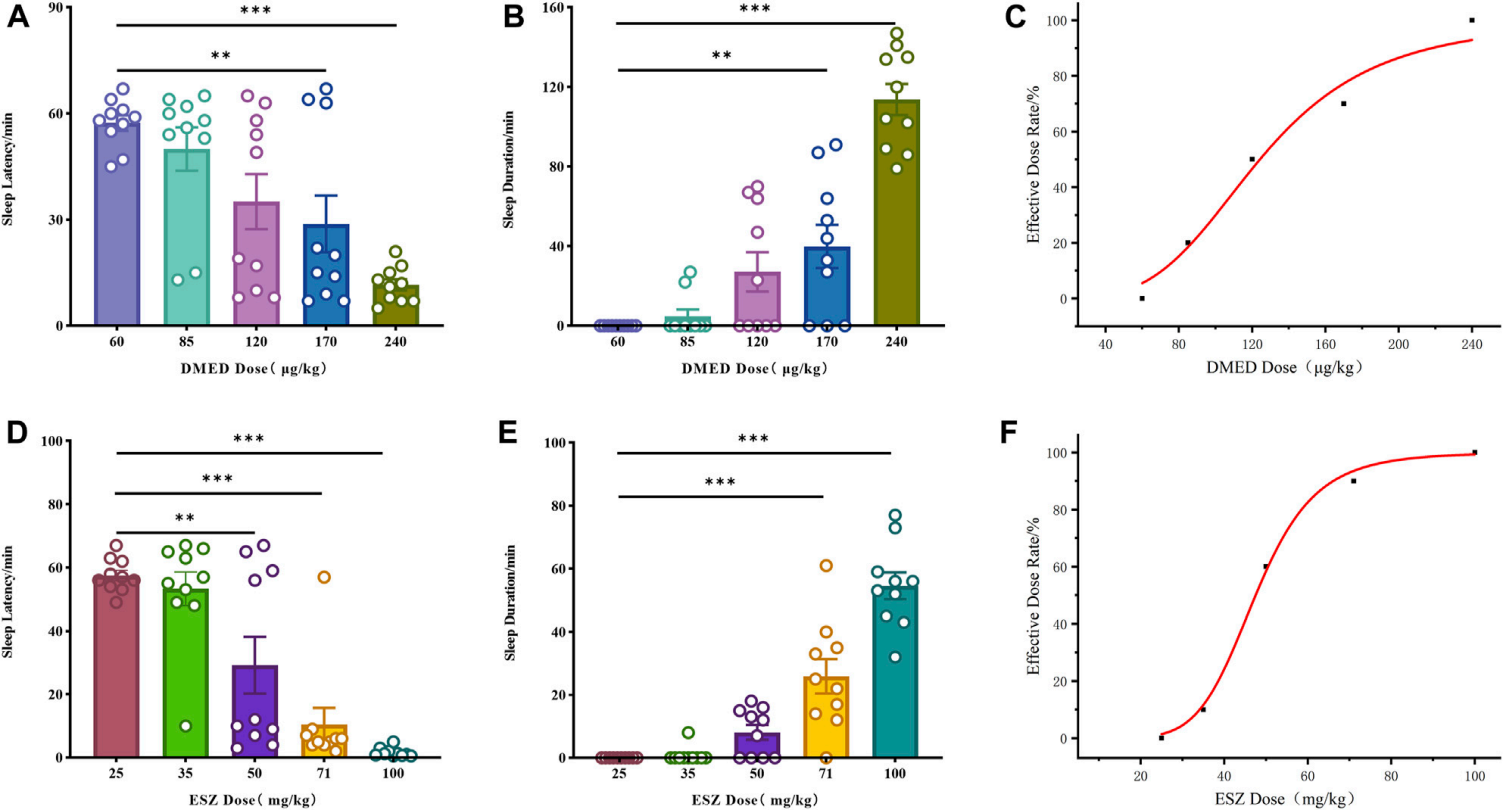
Sleep dose-effect relationships of DMED and ESZ alone. **(A–C)** Effect relationships of sleep latency, sleep duration, and sleep ratio in mice directly induced by intravenous injection of 60, 85, 120, 170, and 240 μg/kg of DMED. As the dose of DMED increased from 60 μg/kg to 240 μg/kg, the sleep latency in mice was significantly shortened, the sleep duration was significantly prolonged, and the sleep ratio was significantly increased. **(D–F)** Effect relationships of sleep latency, sleep duration, and sleep ratio in mice directly induced by intravenous injection of 25, 35, 50, 71, and 100 mg/kg of ESZ. As the dose of ESZ increased from 25 mg/kg to 100 mg/kg, the sleep latency in mice was significantly shortened, the sleep duration was significantly prolonged, and the sleep ratio was significantly increased. Data were expressed as mean ± SEM, *n* = 10. *P < 0.05, **P < 0.01, ***P < 0.001 compared with DMED at 60 μg/kg or ESZ at 25 mg/kg.

The initial results revealed that the minimum doses (ED_100_) of DMED and ESZ required to achieve a 100% hypnotic response rate in mice were 240 μg/kg and 100 mg/kg, respectively, while the maximum doses (ED_0_) resulting in a 0% response rate were 60 μg/kg and 25 mg/kg, respectively. Accordingly, the five subgroup doses for the DMED group were set at 60 μg/kg, 85 μg/kg, 120 μg/kg, 170 μg/kg, and 240 μg/kg, and for the ESZ group, they were 25 mg/kg, 35 mg/kg, 50 mg/kg, 71 mg/kg, and 100 mg/kg. As the dose of DMED increased from 60 μg/kg to 240 μg/kg, the sleep latency in mice was significantly shortened (Figure 1A), the sleep duration was significantly prolonged (Figure 1B), and the sleep ratio was significantly increased (Figure 1C). The hypnotic ED_50_ for DMED was determined to be 124 μg/kg, with a 95% confidence interval ranging from 103.4 μg/kg to 150.1 μg/kg. Similarly, as the dose of ESZ increased from 25 mg/kg to 100 mg/kg, the sleep latency was significantly reduced (Figure 1D), the sleep duration was significantly extended (Figure 1E), and the sleep ratio was significantly elevated (Figure 1F). The hypnotic ED_50_ for ESZ was calculated to be 48 mg/kg, with a 95% CI of 41.2 mg/kg to 56.6 mg/kg.

### 3.2 Interaction between DMED and ESZ combination on sleep in mice

**FIGURE 2:**
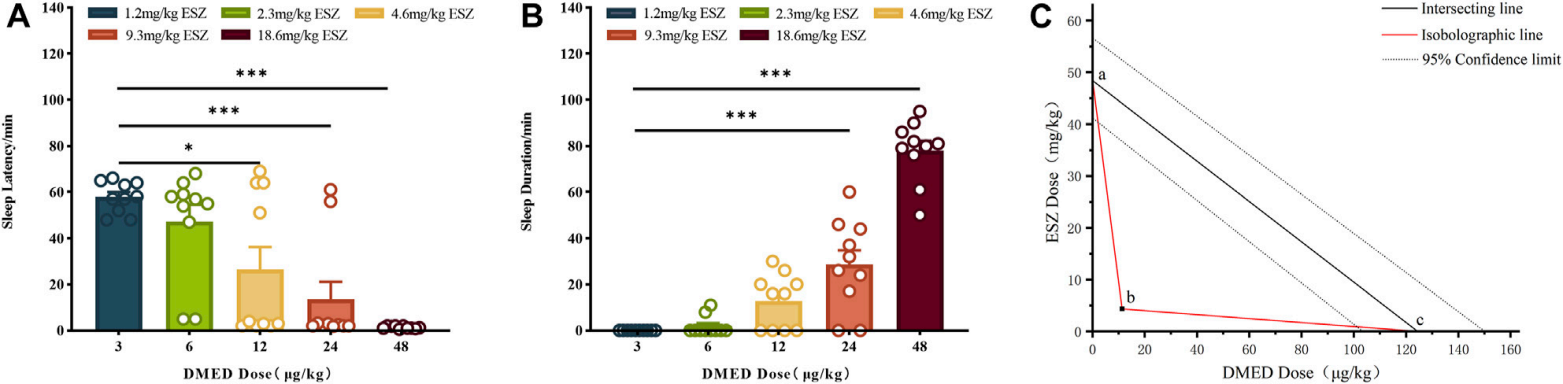
Sleep dose-effect relationship of DMED combined with ESZ. **(A–C)** Sleep latency, sleep duration, and isoradiogram in mice induced by using DMED + ESZ at doses of 3.0 μg/kg + 1.2 mg/kg, 6.0 μg/kg + 2.3 mg/kg, 12.0 μg/kg + 4.6 mg/kg, 24.0 μg/kg + 9.7 mg/kg, and 48.0 μg/kg + 18.6 mg/kg in that order. As the combined dose of DMED and ESZ increased, sleep latency progressively shortened and sleep duration progressively increased. The ED_50_ of DMED was 11.0 μg/kg with a 95% CI of 8.5–13.8 μg/kg, and the ED_50_ of ESZ was 4.4 mg/kg with a 95% CI of 3.1–6.2 mg/kg. The ED_50_ of the combination (point b) fell to the left of the 95% CI of the additive line, confirming a synergistic effect of DMED and ESZ on sleep in mice. Data were expressed as mean ± SEM, *n* = 10, *P < 0.05, **P < 0.01, ***P < 0.001 compared with DMED + ESZ at 3.0 μg/kg +1.2 mg/kg.

DMED and ESZ were co-administered at a fixed dose ratio based on their respective half-effective doses (124:48000, approximately 1:387.1). In previous experiments, the minimum doses (ED_100_) required to achieve a 100% hypnotic response rate in mice were determined to be 48.0 μg/kg for DMED and 18.6 mg/kg for ESZ, while the maximum doses (ED_0_) resulting in a 0% response rate were 3.0 μg/kg for DMED and 1.2 mg/kg for ESZ. Accordingly, the five subgroup doses for the combined DMED+ESZ group were set as follows: 3.0 μg/kg + 1.2 mg/kg, 6.0 μg/kg + 2.3 mg/kg, 12.0 μg/kg + 4.6 mg/kg, 24.0 μg/kg + 9.3 mg/kg, and 48.0 μg/kg + 18.6 mg/kg. As the doses increased, the sleep latency gradually decreased (Figure 2A), and the sleep duration progressively prolonged (Figure 2B). The median hypnotic dose (ED_50_) was achieved at a DMED dose of 11.0 μg/kg, with a 95% CI ranging from 8.5 μg/kg to 13.8 μg/kg, and an ESZ dose of 4.4 mg/kg, with a 95% CI ranging from 3.1 mg/kg to 6.2 mg/kg. In the isobologram (Figure 2C), the coordinates a and c on the axes represent the ED_50_ values of ESZ and DMED when administered alone, respectively, with dashed lines indicating their 95% CIs. The line connecting points a and c represents the additive line. Point b represents the ED_50_ of the combined administration of the two drugs. The nature of the drug interaction is determined by the location of the combined ED_50_ relative to the additive line: if it falls to the left of the additive line, the drugs exhibit a synergistic effect; if it falls to the right, they exhibit an antagonistic effect; and if it lies on the additive line, the effect is additive. Since point b falls to the left of the 95% CI of the additive line, it can be concluded that the combined administration of DMED and ESZ exerts a synergistic effect on sleep induction in mice.

### 3.3 Effects of the combination of DMED and ESZ on the temporal phase of sleep

**FIGURE 3:**
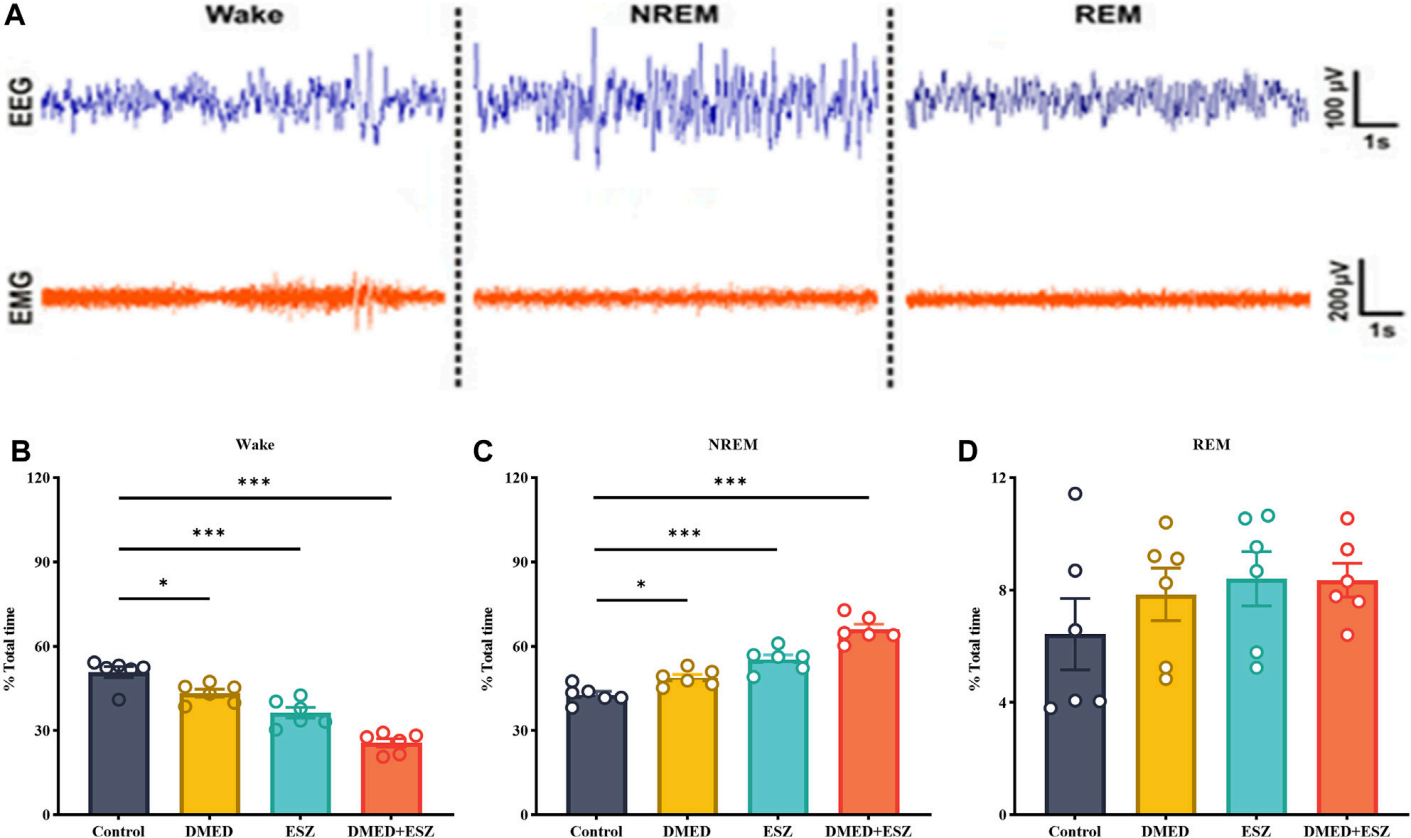
Effect of DMED and ESZ combination on sleep phases. **(A)** Characteristic EEG and EMG patterns of Wake, NREM, and REM phases. The Wake phase is characterized by low-amplitude, high-frequency EEG waves accompanied by significant EMG activity. The NREM phase is marked by high-amplitude, low-frequency EEG waves with minimal EMG activity. The REM phase exhibits low-amplitude, high-frequency EEG waves, and similarly, EMG activity is nearly absent. **(B–D)** Proportions of Wake, NREM, and REM sleep phases. Compared with control group, the wakefulness duration was significantly shortened and the NREM duration was significantly prolonged in the DMED group, ESZ group, and DMED+ESZ group, while there was no significant change in REM duration. Notably, the NREM duration in the DMED+ESZ group was significantly longer than that in the monotherapy groups. Data were expressed as mean ± SEM, *n* = 6, *P < 0.05, **P < 0.01, ***P < 0.001 compared with control.

Based on the aforementioned pharmacodynamic experiments, we selected the combined dose achieving ED100 for further pharmacological studies to investigate the synergistic effects on sleep. Specifically, the DMED group received DMED at 48.0 μg/kg, the ESZ group received ESZ at 18.6 mg/kg, the DMED+ESZ group received both DMED at 48.0 μg/kg and ESZ at 18.6 mg/kg, and the Control group received an equivalent volume of saline. Due to the inverse circadian rhythm of rodents compared to humans, mice are primarily in a sleep or resting state during the daytime. To explore the regulatory effects of DMED and ESZ on daytime sleep-wake cycles under natural circadian conditions, this experiment was conducted during the light phase, corresponding to the resting period of mice. Figure 3A illustrates the characteristic EEG and EMG patterns of Wake, NREM, and REM phases. The Wake phase is characterized by low-amplitude, high-frequency EEG waves accompanied by significant EMG activity. The NREM phase is marked by high-amplitude, low-frequency EEG waves with minimal EMG activity. The REM phase exhibits low-amplitude, high-frequency EEG waves, and similarly, EMG activity is nearly absent. Compared with the Control group, the DMED, ESZ, and DMED+ESZ groups all showed significantly reduced wakefulness (Figure 3B; DMED vs. Control, p < 0.05; ESZ vs. Control, p < 0.001; DMED+ESZ vs. Control, p < 0.001) and significantly prolonged NREM duration (Figure 3C; DMED vs. Control, p < 0.05; ESZ vs. Control, p < 0.001; DMED+ESZ vs. Control, p < 0.001). Notably, the NREM duration in the DMED+ESZ group was significantly longer than that in the monotherapy groups (DMED+ESZ vs. DMED, p < 0.001; DMED+ESZ vs. ESZ, p < 0.001). However, no significant changes were observed in REM duration (Figure 3D; DMED+ESZ vs. Control, P > 0.05). These results are consistent with the isobolographic analysis indicating a synergistic effect of DMED and ESZ on sleep in mice. Furthermore, they suggest that the combination of DMED and ESZ may exert its synergistic effects by prolonging the NREM phase of the sleep architecture.

### 3.4 Regulation of c-fos positive neuronal expression in VLPO and TMN brain regions by the combination of DMED and ESZ

**FIGURE 4:**
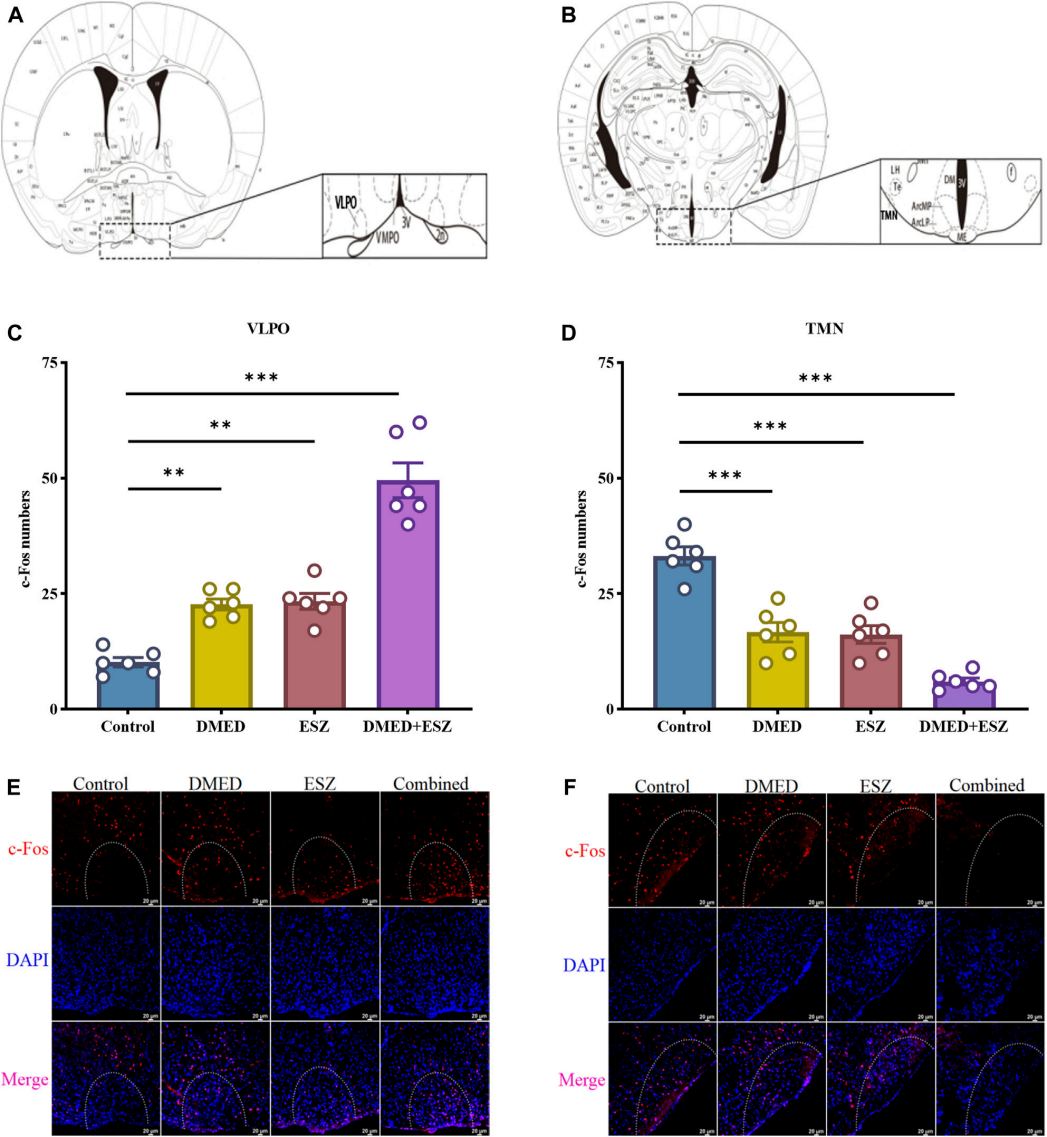
Expression of c-Fos neurons in hypothalamic VLPO and TMN after different treatments. **(A,B)** VLPO brain region, TMN brain region, **(C,D)** c-Fos positive neuron expression in VLPO, c-Fos positive neuron expression in TMN, **(E,F)** IF images of c-Fos positive neurons in VLPO and TMN. Compared with the control group, the number of c-Fos positive neurons in the VLPO increased in the DMED group, ESZ group, and DMED+ESZ group. Notably, the DMED+ESZ combination group showed twice as many c-Fos positive neurons in the VLPO as the monotherapy groups. Contrary to the VLPO results, the number of c-Fos-positive neurons in the TMN decreased in the DMED group, ESZ group, and DMED+ESZ group compared with the control group after drug administration. The reduction in the number of positive neurons in the combination group was 1.6-fold that of the monotherapy groups. Data were expressed as mean ± SEM, *n* = 3, **P < 0.01, ***P < 0.001 compared with control.

The results are shown in Figures 4A,C,E. The control group exhibited the lowest number of c-Fos positive neurons due to the subthreshold nature of low-frequency tonic firing activity in VLPO neurons during natural sleep, which was insufficient to induce c-Fos expression. In contrast, the DMED, ESZ, and DMED + ESZ groups showed an increase in the number of c-Fos positive neurons in the VLPO. Notably, the number of c-Fos positive neurons in the VLPO of the DMED+ESZ group was twice that of the monotherapy groups (P < 0.001). This finding is consistent with the observed sleep state in mice following drug administration. In contrast, the number of c-Fos positive neurons in the TMN significantly decreased after drug administration (Figures 4B,D,F). Compared to the control group, the number of c-Fos positive neurons in the TMN decreased by 50% (P < 0.001), 51% (P < 0.001), and 82% (P < 0.001) in the DMED, ESZ, and DMED+ESZ groups, respectively. Importantly, the reduction in c-Fos positive neurons in the combined treatment group was 1.6 times greater than that in the monotherapy groups (P < 0.01).

### 3.5 Modulation of neurotransmitter levels in the brain by the combination of DMED and ESZ

**FIGURE 5:**
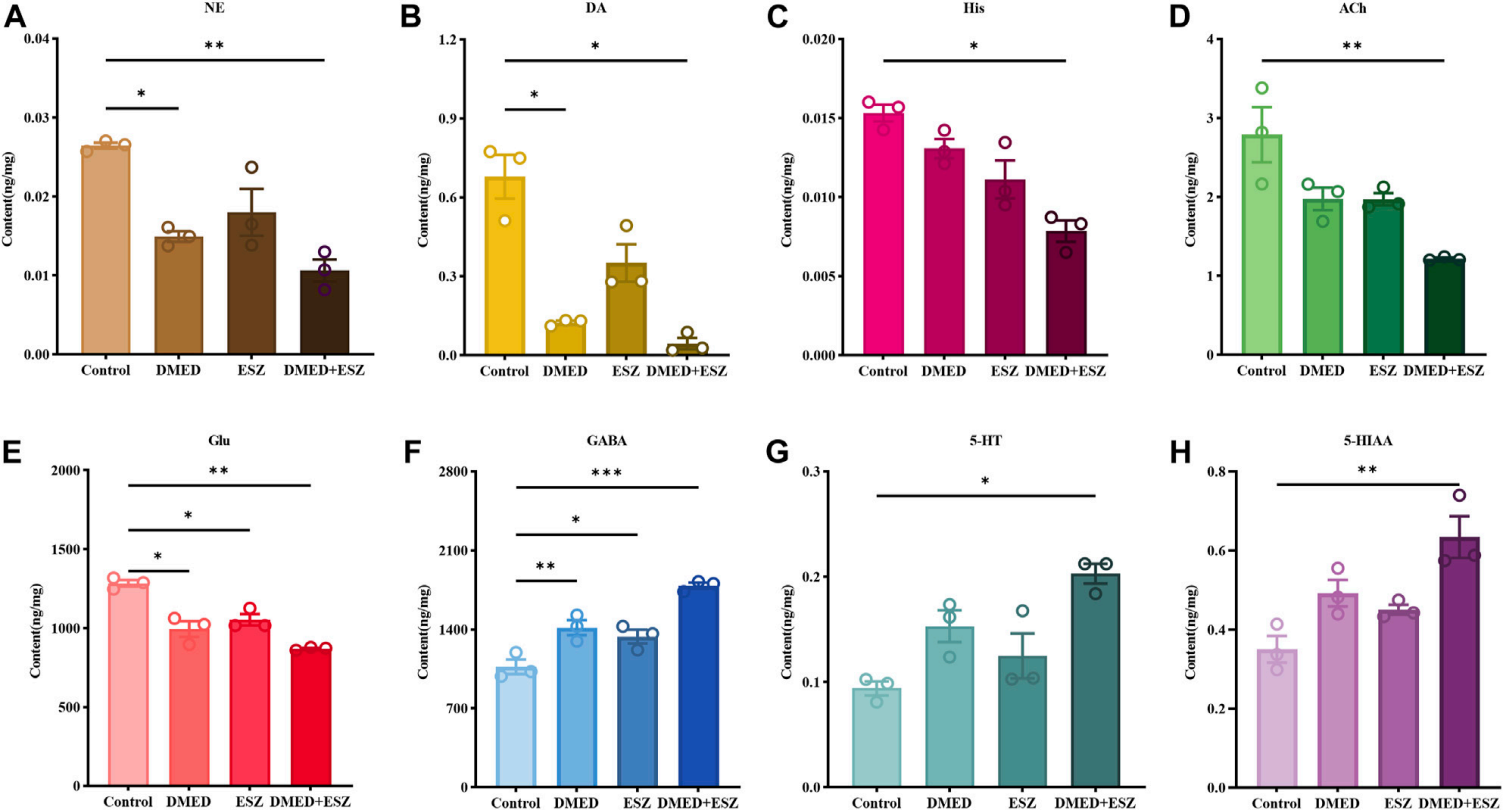
Levels of 8 differential neurotransmitters in the brain. **(A)** NE, **(B)** DA, **(C)** His, **(D)** ACh, **(E)** Glu, **(F)** GABA, **(G)** 5-HT, and **(H)** 5-HIAA. In the DMED+ESZ group, the levels of neurotransmitters DA, NE, His, ACh, and Glu in the brain were significantly decreased, while the levels of GABA, 5-HT, and 5-HIAA were significantly increased. Data were expressed as mean ± SEM, *n* = 3, *P < 0.05, **P < 0.01 compared with control.

The eight neurotransmitters in the brain, NE, DA, His, Ach, Glu, GABA, 5-HT and 5-HIAA were detected and analyzed in this experiment. The experimental results showed that the total ion chromatograms exhibited high resolution and well-defined peaks for all indicators. A linear regression standard curve was plotted with the ratio of the chromatographic peak area of the target standard solution to the internal standard peak area as the y-axis and the concentration as the x-axis. The linearity (*R* ^2^) for all indicators exceeded 0.99, indicating excellent linearity. Quantitative calculations were performed based on the standard curves. As shown in Figure 5, compared to the control group, intravenous administration of DMED and ESZ individually resulted in a decreasing trend in the levels of NE, DA, Ach, Glu, and His in brain tissue, while the levels of 5-HT, 5-HIAA, and GABA showed an increasing trend. After combined administration of DMED and ESZ, the levels of NE (P < 0.05), DA (P < 0.01), His (P < 0.05), Ach (P < 0.01), and Glu (P < 0.01) were significantly reduced, whereas the levels of GABA (P < 0.001), 5-HT (P < 0.05), and 5-HIAA (P < 0.05), were significantly increased. These results suggest that the combined use of DMED and ESZ may exert a synergistic regulatory effect on sleep by altering neurotransmitter levels, specifically by reducing excitatory neurotransmitter levels (including NE, DA, His, ACh and Glu) and increasing inhibitory neurotransmitter levels (including GABA, 5-HT and 5-HIAA), thereby modulating neural activity.

## 4 Discussion

The dose-response relationships for sleep induction by DMED and ESZ administered individually were determined using the LORR experiment. The results demonstrated that both DMED and ESZ significantly shortened sleep latency (Figures 1A,D), prolonged the total sleep time in mice (Figures 1B,E), and the sleep rate increased markedly with higher doses (Figures 1C,F). The ED_50_ for DMED was 124 μg/kg, and for ESZ, it was 48 mg/kg. In the combination drug experiment, the ED_50_ for sleep induction was achieved at a DMED dose of 11.0 μg/kg and an ESZ dose of 4.4 mg/kg. Compared to individual administration, the doses required to achieve the same effect in the combined treatment were reduced by more than 11-fold, indicating a significantly greater effect than simple additive interactions. To further clarify the interaction between DMED and ESZ in combination, we employed the isobolographic analysis, a classical method for evaluating drug interactions. This approach offers the advantage of providing a visual and quantitative assessment of the interaction between two or more drugs, making it particularly valuable in pharmacological research (Tallarida, 2011; Foucquier and Guedj, 2015). The isobolographic analysis revealed that the ED_50_ of the combined treatment fell to the left of the additive line, indicating a synergistic effect between the two drugs in inducing sleep (Figure 2C). To the best of our knowledge, this is the first preclinical study to demonstrate synergistic sleep regulation by combined DMED and ESZ administration in mouse models. Although this specific drug combination has not yet been clinically implemented for human sleep disorders, our findings provide a mechanistic foundation for translational research. From a clinical perspective, such combination therapy may substantially reduce required doses of individual agents, thereby minimizing side effects associated with high-dose monotherapy. Furthermore, for patients with sleep disorders refractory to monotherapy, the DMED-ESZ combination constitutes a novel therapeutic strategy that may enhance clinical efficacy and improve sleep quality.

The sleep-wake state is mainly divided into three states: NREM, REM, and Wake, each characterized by distinct electroencephalogram (EEG) features. During NREM sleep, EMG signals nearly disappear, the overall EEG frequency slows down, the amplitude increases, and Delta waves become significantly enhanced. During REM sleep, EMG signals also nearly disappear, but the overall EEG frequency increases, the amplitude decreases, and Theta waves become notably enhanced. In contrast, during the Wake phase, EMG signals are significantly enhanced, the overall EEG frequency increases, and the amplitude decreases (Scammell et al., 2017; Claar et al., 2023). Based on the EEG/EMG monitoring results, the combined administration of DMED and ESZ significantly reduced the duration of wakefulness (Figure 3A), prolonged the duration of NREM sleep (Figure 3B), and this effect was markedly superior to that of individual drug administration. This aligns with previous studies indicating that hypnotic drugs typically alter total sleep time and NREM sleep rather than REM sleep (Bajwa and Kulshrestha, 2013; Hajiaghaee et al., 2016; Yoon and Cho, 2018; Kim et al., 2019; Shi et al., 2019). From a functional perspective, NREM sleep plays a critical role in bodily recovery, energy conservation, and memory consolidation (Sun et al., 2020). The extension of NREM sleep by the combined treatment suggests its significant potential in promoting physiological maintenance and enhancement. Adequate NREM sleep facilitates protein synthesis and the repair of damaged tissues, which are essential for growth, development, and daily recovery (Tononi and Cirelli, 2014). This indicates that the combined treatment may improve overall sleep quality by enhancing the physiological functions associated with NREM sleep. Furthermore, the prolongation of NREM sleep may positively impact cognitive functions. DMED has been shown to protect neurocognition by reducing interleukin (IL)-6 and IL-1β in the hippocampus (Wang et al., 2019; Mei et al., 2021). It also improves cognitive function by reducing inflammation via α_2_-AR-mediated decreases in IL-1β and NF-κB levels (Li et al., 2020). ESZ can effectively improve the sleep quality in patients with sleep disorders, reduce the number of nocturnal awakenings, and thereby enhance cognitive function (Huo et al., 2022). The significant increase in NREM sleep in this study suggests that the combined treatment may improve cognitive ability in mice. This finding provides a new theoretical basis for the clinical application of combined drug therapy. In the sleep cycle, NREM and REM sleep alternate, and the balance between the two is crucial for maintaining normal sleep and physiological functions. Although the combined treatment in this study did not significantly affect REM sleep (Figure 3C), the extension of NREM sleep may indirectly influence the functions related to REM sleep. Future studies could further explore the effects of combined treatment on the alternating patterns of NREM and REM sleep throughout the sleep cycle.

The c-Fos protein, a product of the immediate-early gene c-fos, serves as a marker of neuronal activity and is widely used to study functional anatomy and neuronal activation states in the nervous system (Zant et al., 2016). As studies have indicated, the VLPO and TMN play central and well-defined roles in regulating the sleep-wake cycle (Chung et al., 2017; Scammell et al., 2019). By focusing on the VLPO and TMN, we can target the core components of the sleep-wake regulatory network. In this study, using c-Fos immunofluorescence staining, we investigated the effects of combined DMED and ESZ administration on neuronal activity in the VLPO and TMN brain regions. VLPO neurons primarily release inhibitory neurotransmitters, GABA and glycine. Existing studies have comprehensively demonstrated the central role of the VLPO in sleep initiation and maintenance through various perspectives, including neurotransmitter regulation (Gaus et al., 2002), environmental influences (Gong et al., 2000), and circuit mechanisms (Cirelli and Tononi, 2000), as evidenced by c-Fos immunohistochemistry. In contrast to the VLPO, the TMN is a key nucleus for maintaining wakefulness and is the sole source of histaminergic (His) neurons (Yu et al., 2014). In 2005, Saper and colleagues proposed the “flip-flop switch model” for sleep-wake transitions, which hypothesizes that sleep-promoting neurons in the VLPO and wake-promoting neurons in the TMN mutually inhibit each other to facilitate sleep-wake transitions (Saper et al., 2005). Our results showed that the combined treatment increased the number of c-Fos positive neurons in the VLPO (Figures 4C,E) while decreasing the number of c-Fos positive neurons in the TMN (Figures 4D,F). These findings align with the “flip-flop switch model,” further supporting the hypothesis that the combined administration of DMED and ESZ promotes sleep by modulating neuronal activity in these two brain regions. Additionally, the effect of sleep-inducing drugs on the VLPO is closely associated with the prolongation of NREM sleep. During NREM sleep, VLPO neuronal activity increases, leading to elevated expression of c-Fos protein, the product of the c-fos gene. Studies using optogenetics have shown that inhibiting the excitability of histaminergic neurons in the TMN during wakefulness can induce NREM sleep but not REM sleep (Venner et al., 2016; Yamashita and Yamanaka, 2017), which is consistent with our experimental results showing that the combined treatment suppresses REM sleep while enhancing NREM sleep.

The regulation of sleep and wakefulness is widely recognized to be related to the levels of corresponding neurotransmitters (Holst and Landolt, 2018). And NE, DA, Ach, Glu, and His as excitatory neurotransmitters play a role in initiating and maintaining wakefulness in the sleep-wake cycle, and 5-HT, 5-HIAA, and GABA as inhibitory neurotransmitters can induce and maintain sleep state. The results of the present study showed that the combination of DMED and ESZ significantly decreased the levels of DA, NE, Ach, Glu, and His, while increasing the levels of 5-HT, 5-HIAA, and GABA, suggesting that the combination synergistically promotes the molecular mechanism of sleep through the bi-directional regulation of excitatory-inhibitory neurotransmitter balance. DMED, as an α2-AR agonist, negatively feeds back by inhibiting the activity of NEergic neurons in the LC and reduces NE release (Bajwa and Kulshrestha, 2013), while ESZ may indirectly reduce NE levels by enhancing GABAergic inhibition. NE levels were significantly reduced after the combination in this study (Figure 5A), suggesting that the two drugs may reduce arousal drive by synergistically inhibiting the LC-NE pathway. The dopaminergic neurons are mainly located in the VTA and substantia nigra compacta (SNc) of the midbrain, with extensive fiber connections to the sleep-wake brain regions, and their dopaminergic signals projected to the forebrain play a key role in maintaining arousal and motivational behaviors (Montero et al., 2021). DA levels were significantly reduced in the present study (Figure 5B), suggesting that the combination may reduce arousal drive by inhibiting dopaminergic neuronal activity in the VTA and SNc. Noradrenergic neurons project to the VTA via the medial forebrain bundle (MFB). During wakefulness, VTA dopaminergic neurons exhibit activation states, whereas inhibition of these neurons promotes sleep (Eban-Rothschild et al., 2016). DMED, through its suppression of noradrenergic signaling, attenuates excitatory drive to VTA-DA neurons, thereby reducing dopamine (DA) release. ESZ, a GABA-A receptor agonist, exerts its effects by potentiating GABAergic inhibitory signaling. DA neurons in the VTA and SNc receive GABAergic projections from the ventral pallidum and nucleus accumbens, which directly suppress DA neuronal activity. Concurrently, enhanced GABAergic tone inhibits cortical and limbic dopaminergic systems, further reducing DA release (Tan et al., 2012). His is secreted only by histaminergic neurons in the TMN brain region, whose nerve fibers project widely throughout the brain and are essential for maintaining arousal (Scammell et al., 2019), and the reduction in His levels after the combination is consistent with the reduction in c-Fos positive neurons in the TMN (Figure 5C). This further supports the finding that inhibition of TMN activity is an important mechanism for sleep promotion. Acetylcholinergic neurons are mainly located in the LDT and PPT of the brainstem. Acetylcholine (ACh), which is involved in sleep regulation, is released from the terminals of these neurons and widely projects to the cerebral cortex, amygdala, hippocampus, thalamus, brainstem, and midbrain to promote arousal (Jones, 2020). Glu drives arousal in the thalamo-cortical loop (Dash et al., 2009). The significant decrease in Ach and Glu after the combination suggests that the two drugs may synergistically attenuate arousal signaling through inhibition of thalamo-cortical glutamatergic transmission and basal forebrain acetylcholinergic activity (Figures 5D,E). GABA, as a major inhibitory neurotransmitter in the central nervous system (CNS), mediates sleep via GABA-A receptors in areas such as the VLPO and basal forebrain (Scammell et al., 2017; Cai et al., 2023), ESZ acts as a GABA-A receptor agonist and directly enhances GABAergic inhibition, whereas DMED may have a synergistic effect by inhibiting noradrenergic release and deregulating inhibition of GABAergic neurons. The GABA content in this study was significantly elevated after the combination (Figure 5F), suggesting that the two drugs amplify GABAergic signaling through a dual mechanism (direct activation of receptors and deregulation of upstream inhibition). 5-HT neurons in the DRN exhibit complex modulatory roles in sleep-wake transitions, with their primary metabolite being 5-HIAA (Oikonomou et al., 2019). Accumulation of 5-HT may promote sleep through activation of 5-HT2A receptors. The elevation of 5-HT and 5-HIAA in the present study may reflect the modulation of the DRN-5-HT system by the combination of drugs (Figures 5G,H), but the exact mechanism needs to be further investigated. The initiation of REM sleep is associated with suppression of 5-HT and NE neurons, potentially mediated by activation of the lateral habenula (LHb) (Zhao et al., 2015; Takahashi et al., 2022). In this study, the sustained inhibition of NE may establish permissive neurochemical conditions for REM sleep. Notably, 5-HT has been demonstrated to activate the claustrum while inhibiting theta rhythm-promoting brain regions (Hajos et al., 2003; Nuñez and Buño, 2021). This dual action aligns with the insignificant REM sleep changes observed in the current study, potentially explaining why REM sleep remained unaffected.

## 5 Conclusion

In summary, this study reveals for the first time that the combination of DMED and ESZ has a synergistic effect in regulating sleep in mice, and elucidates the potential mechanism of the synergistic effect in terms of changes in sleep phases, c-Fos protein expression, and neurotransmitter content. The finding provides a significant theoretical foundation for the clinical application of combination therapy in treating sleep disorders and lays the groundwork for the development of novel sleep-regulating drugs.

## Data Availability

The data that support the findings of this study will be available upon reasonable request to the corresponding authors.
