# Correction: Synergistic effects of combined hypnotic drugs on sleep in mice

**DOI:** 10.3389/fphar.2025.1672624

**Published:** 2025-08-14

**Authors:** Frontiers Production Office

**Affiliations:** Frontiers Media SA, Lausanne, Switzerland

**Keywords:** sleep, hypnotics, dexmedetomidine, eszopiclone, combination, synergistic effects

In the published article the affiliation of the reviewer Lucio Antonio Ramos-Chávez was incorrectly inserted as “Trinity College Dublin, Ireland”. The correct affiliation is “Instituto Nacional de Psiquiatría Ramón de la Fuente, Mexico.”

The original version of this article has been updated.

